# Diagnosis of Rickettsioses from Eschar Swab Samples, Algeria

**DOI:** 10.3201/eid1710.110332

**Published:** 2011-10

**Authors:** Nadjet Mouffok, Cristina Socolovschi, Anwar Benabdellah, Aurelie Renvoisé, Philippe Parola, Didier Raoult

**Affiliations:** Centre Hospitalier Universitaire d’Oran, Oran, Algeria (N. Mouffok, A. Benabdella);; Université de la Méditerranée, Marseille, France (C. Socolovschi, A. Renvoisé, P. Parola, D. Raoult)

**Keywords:** Eschar swab, eschar, R. conorii conorii, rickettsiosis, diagnosis, point of care, rickettsia, Algeria, letter

**To the Editor:** Tick-borne rickettsioses are zoonoses caused by intracellular bacteria belonging to the spotted fever group rickettsiae (*1*). The main clinical signs are high fever, maculopapular rash, and an inoculation eschar at the site of the tick bite (corresponding to the portal of entry of rickettsiae into the host). Recently, several rickettsioses were diagnosed by using swab samples from skin lesions (*2**,**3*). In an animal model, as long as eschars were present, rickettsial DNA was detected (*2*). Our aim was to evaluate the advantage of skin swab samples for diagnosis of rickettsial diseases in a country where rickettsioses are endemic (*4*).

From July 2009 through October 2010, a total of 39 patients in the infectious disease department of Oran Teaching Hospital, Algeria (27 men, 12 women; median age 46.5 years) were included in a prospective study. The mean (± SD) interval between onset of lesions and consultation was 7 ± 1.8 days. Underlying conditions were present in 13 (33%) patients: diabetes (4 patients, 10.2%), hypertension (2 patients, 5%), chronic renal failure (1 patient, 2.5%), cervical cancer (1 patient, 2.5%), bronchial cancer (1 patient, 2.5%), and tobacco consumption (6 patients, 15%). Fever and generalized maculopapular rash (also on palms and soles) were found for 38 (97.4%) patients, including 5 (12.8%) with purpuric rash. One (2.5%) patient had 2 eschars on the back. Eschars were frontal (1 [2.5%] patient), cervical (3 [7%]), axillary (4 [10.2%]), on the back (6 [15%]), on the abdomen (2 [5%)], on the thorax (1 [2.5%]), inguinal (6 [15%]), on the leg (8 [20.5%]), on the arm (1 [2.5%]), on the buttock (2 [5%]), on the breast (1 [2.5%]), on the nipple (1 [2.5%]), on the penis (1 [2.5%]), and on the scrotum (2 [5%]). Conjunctivitis was reported for 24 (61.5%) patients and myalgia for 34 (87.8%). Lymphadenopathy was found near the eschar for 8 (20.5%) patients. Antimicrobial drug therapy (doxycycline 200 mg 1×/d) for 3.5 ± 1.4 days was empirically prescribed for all case-patients before diagnoses were confirmed.

A dry sterile swab (Copan, Brescia, Italy) sample was collected from the inoculation eschar of each patient by the same person (N.M.). Two patients had 2 swab samples collected from the same eschar. Only 1 swab sample was collected from the patient with 2 eschars. Scabs were removed from eschars before swabbing. The swabs, while being rotated vigorously, were directed to the base of the eschar at a 50°–60° angle for 5–6 times. For 4 patients, an eschar biopsy sample was collected under sterile conditions. Swabs were then placed back in their tubes and stored at –20°C before transportation to Unité des Rickettsies, Marseille, France.

In the laboratory, each sample was placed in 2 mL of culture medium. DNA was extracted from 200 µL of solution of eschar swab or skin biopsy samples by using the QIAamp DNA Mini Kit (QIAGEN, Hilden, Germany) according to the manufacturer’s instructions, with a final elution volume of 100 µL. We used quantitative real-time PCR (qPCR) to determine quality of DNA extraction and level of housekeeping gene coding for β-actin (*5*) and to detect rickettsiae (*2**,**5*). Mean (± SD) cycle threshold (C_t_) value of the β-actin gene for all swab samples was 24.9 (± 2.7). Of 41 swab samples, 26 were positive for rickettsial DNA (63.4%) by qPCR targeting the RC0338 gene (*5*); mean C_t_ value was 33.99 (± 2.15). Specific *Rickettsia conorii conorii* qPCR, targeting the putative acetyltransferase gene (*2*), had positive results for 25 (64%) patients; C_t_ values ranged from 31.02 to 38.63. This sensitivity is comparable to that of PCRs for detecting *R. conorii*, the agent of Mediterranean spotted fever, on skin biopsy samples (*4**,**6*). Mean C_t_ value for β-actin gene amplification of *R. conorii conorii*–positive swab samples was 23.84 (± 2.19), significantly lower than that for *R. conorii conorii*–negative samples (26.84 ± 2.72; p = 0.0003). Of 4 patients for whom swab and skin biopsy samples were available, 3 had positive results.

Opinions of health care providers and patients were evaluated by using standard questionnaires (Table). Most health professionals preferred collecting swab samples over biopsy samples for patients and for themselves (46 vs. 5 and 57 vs. 2, respectively; p = 0.0001). Patients from France and Algeria also preferred having a swab sample taken over a skin biopsy sample (43 vs. 7; p = 0.0001). Statistical analyses were conducted by using GraphPadPrism version 2.0 (www.graphpad.com/prism/Prism.htm) to p<0.5.

**Table:** Acceptability of swab or biopsy samples from tick-bite eschars, Oran Teaching Hospital, Oran, Algeria, July 2009–October 2010

Question	Answer, no. (%)*			
	Eschar swab sample	Skin biopsy sample	Both samples	Nothing
For medical or biologist practitioners, n = 78				
In the case that your patient has a “tache noire,” what would you do to confirm the diagnosis?	46 (59)	5 (6.4)	22 (28)	5 (6.4)
If you have a “tache noire,” what do you do to confirm the diagnosis?	57 (73)	2 (2.5)	9 (11.5)	9 (11.5)
For patients, n = 58				
In the case of inoculation eschar, which kind of medical procedure would you prefer your medical doctor to conduct?	43 (74)	78 (12)	5 (8.6)	3 (5.2)

*p = 0.0001 for eschar swab sample vs. skin biopsy sample; p value not available for other parameters.

Swabbing an eschar is a rapid and simple technique that can be easily performed without risk for the side effects associated with biopsy sampling. Insufficient material taken during swabbing, evidenced by high C_t_ values of β-actin, results in low rickettsial load, explaining the false-negative results. This test can be used at the bedside or in an outpatient clinic and could be useful for epidemiologic and clinical studies. Because qPCR results can be obtained <4 hours after sampling, this technique might be useful for point-of-care diagnosis.
