# Correction: Vol. 17, No. 10

**DOI:** 10.3201/eid3004.C23004

**Published:** 2024-04

**Keywords:** errata, erratum, correction

The name of author Anwar Benabdellah was omitted in Diagnosis of Rickettsioses from Eschar Swab Samples, Algeria (N. Mouffok et al.). The article has been corrected online (https://wwwnc.cdc.gov/eid/article/17/10/11-0332_article).

